# Ethics and antibiotic resistance

**DOI:** 10.1093/bmb/ldab030

**Published:** 2022-02-05

**Authors:** Euzebiusz Jamrozik, George S Heriot

**Affiliations:** The Ethox Centre and Wellcome Centre for Ethics and Humanities, Nuffield Department of Population Health, University of Oxford. Old Road Campus, Oxford OX3 7LF, UK; Monash Bioethics Centre, Monash University, Wellington Rd, Clayton, 3800, Victoria, Australia; Department of Medicine, Royal Melbourne Hospital, University of Melbourne, 300 Grattan St, Parkville, 3050, Victoria, Australia; Department of Medicine, Royal Melbourne Hospital, University of Melbourne, 300 Grattan St, Parkville, 3050, Victoria, Australia

**Keywords:** drug resistance, antimicrobial resistance, bioethics, justice, tragedy of the commons, stewardship

## Abstract

**Introduction or background:**

Antibiotic resistance raises ethical issues due to the severe and inequitably distributed consequences caused by individual actions and policies.

**Sources of data:**

Synthesis of ethical, scientific and clinical literature.

**Areas of agreement:**

Ethical analyses have focused on the moral responsibilities of patients to complete antibiotic courses, resistance as a tragedy of the commons and attempts to limit use through antibiotic stewardship.

**Areas of controversy:**

Each of these analyses has significant limitations and can result in self-defeating or overly narrow implications for policy.

**Growing points:**

More complex analyses focus on ethical implications of ubiquitous asymptomatic carriage of resistant bacteria, non-linear outcomes within and between patients over time and global variation in resistant disease burdens.

**Areas timely for developing research:**

Neglected topics include the harms of antibiotic use, including off-target effects on the human microbiome, and the lack of evidence guiding most antibiotic prescription decisions.

## Introduction

It is widely acknowledged that the development of resistance by human bacterial pathogens to pharmaceutical antimicrobials due to their use (acquired antibiotic resistance or acquired ABR) poses a major threat to global public health. Recent data suggest that over 1 million deaths per year are attributable to resistant bacterial infections^1^

Much of this disease burden is concentrated in low- and middle-income countries (LMICs) where surveillance for resistance is often incomplete.^2^ For example, a 2016 estimate suggested that over 200 000 deaths of newborns each year, primarily in LMICs, are caused by resistant bacterial sepsis, and many of these cases would not have been counted in the global estimate above.

Acquired ABR is an important topic for ethical analysis since the consequences for human health and well-being are often severe (and conversely, the benefits of effective antibiotics are enormous), the harms of resistant infection are often inequitably distributed and human actions are major contributors to the problem.^3^ Making and implementing policy to address drug resistance involves balancing multiple ethical values, as well as multiple types of harms and benefits.^2^^,^^3^

In this article we review key concepts, scientific data and ethical issues relevant to clinically significant acquired ABR in common bacterial infections in humans, outlining the advantages and limitations of common conceptual and policy approaches to ABR. In light of these analyses, we discuss appropriate antibiotic use policies in humans, public health interventions among asymptomatic carriers of resistant bacteria and public health priorities in different global settings. We conclude by identifying avenues for future research and policy development related to acquired ABR, noting previous work on ethical issues related to some other types of non-bacterial antimicrobial resistance (e.g. anti-tubercular drug resistance,^4^ antiretroviral resistance,^5^ anti-malarial resistance,^6^ as well as antibiotic use in animals^7^).

## Background

This narrative review will approach the topic of antibiotic resistance from the perspective of pluralistic public health ethics, acknowledging that ethical evaluations and policy decisions involve trade-offs between important values, primarily health (or utility, well-being, benefits and harms, etc.), fairness (or equality, equity, justice, etc.) and freedom (or liberty).^8^ Before embarking on a tour of the ethical issues related to acquired ABR, it is important to review the biology and epidemiology of the problem, as scientific publications often involve implicitly ethical analysis, whether or not the moral issues or values at stake are made explicit, which we will seek to do throughout this review. Moreover, ethical analyses of ABR should arguably be informed by relevant scientific concepts and facts about, for example, prevalence and disease burden, which can help to focus ethical analyses on the most common or harmful problems. Although this review focuses on antibiotic resistance in humans, we note that problems related to resistant bacteria involve multiple species and sectors, and at this broader level, ABR may be best characterized as a One Health problem.^9^^,^^10^

Bacteria with the potential to cause disease in humans are ubiquitous: in the human microbiome that contains more bacterial organisms than cells of the host organism,^11^ in the microbiomes of other humans with whom each person is in contact and in the external environment. The majority of the global burden of human bacterial disease is linked to organisms that are carried by healthy individuals for long periods of time,^12^ often asymptomatically, either as transient colonisers or as part of the normal human microbiome.

The use of antibiotics necessarily exposes this microbial ecosystem to variable concentrations (at different niches within the body) of compounds that are variably lethal to different bacterial species (or subpopulations within a species). After exposure, pathogens can acquire additional resistance through selection for resistant mutants, activation of latent resistance mechanisms or acquisition of mobile resistance determinants from other species,^7^ which can be enhanced by antibiotic pressure.^13^ The clearing of niches for more resistant pathogens is common and likewise involves antibiotic pressure increasing the overall prevalence of resistance to any given antibiotic. Bacteria can also be exposed to antibiotics in the environment, for example in human effluent, clinical waste, and in agricultural or veterinary settings.^7^ In addition, people not exposed to antibiotic therapy can acquire resistant organisms directly through contact with infected or colonized people, animals or other environmental reservoirs.

Although the magnitude of the benefit of living in the antibiotic era is hard to under-estimate,^2^ the use of antibiotic compounds (among other human actions) can result in, or at least accelerate, the development of acquired ABR in pathogens that can then go on to cause disease. This tension between the benefits of having effective antibiotics available to treat infectious diseases and the loss of availability through their use is the core dilemma that ethicists, among others, have sought to address have sought to address.

## Fleming and ‘finishing the course’

Concerns about acquired ABR arose very early in the antibiotic era. Alexander Fleming provided an early formulation of the problem in 1945, when he claimed that: ‘the greatest possibility of evil…is the use of too-small doses [of penicillin], so that, instead of clearing up the infection, the microbes are educated to resist penicillin and a host of [resistant] organisms is bred out which can be passed on to other individuals and perhaps from there to others until they reach someone who gets a septicemia or a pneumonia which penicillin cannot save. In such a case the thoughtless person playing with penicillin treatment is morally responsible for the death of the man who finally succumbs to infection with the penicillin-resistant organism’.^14^

This compelling description of origin of acquired ABR through ‘inadequate’ treatment of an ‘eradicable’ pathogen that is ‘unavoidably’ transmitted directly between neighbouring individuals has been widely influential in discussions of acquired ABR ever since (despite being accurate only in a narrow set of situations, ironically not including the streptococcal pharyngitis used as an example by Fleming). It may be tempting to think that patients failing to finish the course is one of the most important ethical issues related to ABR. Indeed, the specious simplicity of Fleming’s parable allows a line of moral culpability to be drawn directly from the original negligent prescriber (or non-adherent patient) to the victim denied effective antibiotic therapy. The obvious solutions are maximalist and technical: larger doses, longer courses of therapy and perhaps a broader spectrum agent to ensure all potential pathogens are ‘covered’, followed by the development of new antibiotic drugs (or other technological fixes) when old ones fail.^15^

While it is true that under-dosing of (or otherwise inappropriate) antibiotic therapy can result in acquired ABR in the infecting pathogen (for example, *Neisseria gonorrhoeae* and cephalosporins^16^), the vast majority of antibiotic resistance in humans caused by clinical use of antibiotics results instead from the off-target effects of antibiotic therapy on the modifiable but non-eradicable wider microbiome (sometimes termed bystander selection).^16^^,^^17^ The magnitude of these off-target effects is necessarily larger with broader-spectrum agents and also seems to increase with longer durations of antibiotic therapy,^18^ both of which increase harms for the original patient.^19^ Higher concentrations of antibiotics do appear to reduce the generation of resistant mutants in relatively low-inoculum *in vitro* monocultures,^20^ but this may not apply to more complex *in vitro* models^21^ or actual clinical trials,^22^ let alone clinical practice. Fleming’s maximalist solution, while simple and free from ethical trade-offs (given sufficient supply), is insufficient and, in reality, often unhelpful in attempts to limit the prevalence of and harm due to acquired ABR.

## The tragedy of the commons

The recognition that antibiotic use increases acquired ABR, and thereby may reduce the availability of effective antibiotic therapy for all, led to the formulation that antibiotic use is analogous to one type of collective action problem known as the tragedy of the commons.^23–30^ Standard commons tragedies such as overfishing involve a common resource (e.g. fish stocks) where each person who benefits from the resource has an incentive to use more of the resource (e.g. catch more fish) with the tragic outcome being the depletion of the common resource to the point where every person is made worse off.^29^^,^^31^ An important implication of framing resistance as a commons tragedy is that this implies that responses should arguably include, and perhaps even be primarily focused on, regulating human behaviour—with the key behaviour in this case being consumption of antibiotics.

On the one hand, framing antibiotic resistance as a tragedy of the commons has several advantages. It can help individuals and institutions to (i) understand the resource at stake (often conceptualized along the lines of ‘access to effective antibiotics’),^31^ (ii) identify the limits of certain types of purported solutions to the problem (e.g. that new drug discovery is unlikely to be a panacea for drug-resistant bacterial infection^15^), (iii) consider the appropriate justification(s) of regulating access to the common resource.^23^

On the other hand, antibiotic resistance is disanalogous with standard commons tragedies in multiple ways. First, an increase in an individual’s consumption of antibiotics does not result in progressively greater benefits. Although antibiotics may be highly beneficial in the context of active infection, they offer little if any benefit to healthy individuals, and prophylactic or immunomodulatory use in those with underlying conditions is helpful only in a very narrow set of circumstances. The maximal expected benefit of any antibiotic treatment occurs when therapy is targeted against a definitively diagnosed infection, but multiple uncertainties erode this benefit in real-world situations. These include (i) diagnostic uncertainty, as the cause of a patient’s illness may not be due to bacterial infection^32^^,^^33^; (ii) therapeutic uncertainty, as not all patients with a bacterial infection benefit from antibiotic therapy^34^; (ii) microbiological uncertainty, microbiological uncertainty, as pathogens are not uniformly susceptible to all available antibiotic options (and this information may not be available at the time treatment in initiated).

Second, in contrast to these diminishing benefits, antibiotic therapy is accompanied by a reasonably fixed range of adverse effects. As well as acquired ABR, these include allergic reactions, organ toxicities, superinfections (such as mucosal candidiasis or toxigenic *Clostridioides difficile*) and other dysbiosis-related risks such as an increased incidence of inflammatory bowel disease^35^ and even obesity.^36^ Therefore, the ratio of benefits to risks of antibiotic therapy declines with increasingly eager use and is entirely inverted in gratuitous overuse. Although gratuitous overuse might deplete the (whole population) commons of antibiotic effectiveness to some degree, gratuitous individual users will be the primary sufferers of these harms of overuse.

Third, although resistant bacteria can be transmitted between people, the degree to which this occurs can be significantly reduced by public health measures including sanitation, and by specific infection control measures in healthcare and other higher-risk settings.^33^ Interventions to reduce antimicrobial consumption (and thereby generation of resistant pathogens) in high-risk settings are significantly more effective at reducing the prevalence of resistant pathogens when they are combined with interventions that prevent transmission between individuals.^37^ Conversely, exposure to locations with a higher prevalence of acquired ABR (including during hospital admission) can introduce these resistant organisms to low-prevalence populations even in the absence of local antibiotic consumption,^2^ and sociodemographic differences appear to result in a different strength of association between antibiotic consumption and the development of acquired ABR at the ecological level.^38^ The probability of transmission influences the degree to which the ‘commons’ is depleted above and beyond any use of antibiotics that contributed to the initial development of resistance.

Fourth, because transmission between individuals is incomplete, the harms of resistant infection are ‘concentrated’ among patients exposed to antibiotic therapy, rather than ‘shared evenly’ across all who might stand to benefit from the common resource of effective antimicrobials.^17^ Individuals recently exposed to antibiotics are much more likely to harbour resistant organisms than controls,^39^^,^^40^ and antibiotic-resistant infections are most likely to occur in those previously exposed.^18^ Strong associations between individuals’ antibiotic consumption and colonization or infection with resistant isolates can be identified even when associations at the ecological (‘commons’) level are absent.^41^^,^^42^ The implication of this observation is that the ‘commons’ concept may be more applicable within one individual over time than across a population in settings or for organisms where transmissibility is low. Antibiotic use is not a ‘free ride’ (each use involves risk, these risks are concentrated among users), and patients who use disproportionately large amounts of antibiotics are not ‘free riding’ on the temperance of others insofar as transmission of resistance is limited and the prevalence of resistance is at least partly determined by factors other than antibiotic use.

Nevertheless, discussing resistance as a tragedy of the commons might at least help to support some useful policy approaches, for example, environmental and infection control policies that reduce transmission (a form of harm capitation independent of antibiotic use) as well as regulation of antibiotic consumption. However, as we describe in the next sections, the governance of antibiotic use through idealized prescription guidelines and other stewardship interventions faces multiple real-world challenges arising from the many agents and interests involved in practice.

## Prescribers, agents and conflicts of interest

### Multiple agency problems

Another significant departure from the standard ‘commons’ formulation is the fact that, in most healthcare systems, patients do not have unmediated access to antibiotics. When the central role of the prescriber is added to the (assumed) collective action challenge of the tragedy of the commons, the result is a ‘dual agent’ problem first recognized in medical economic analyses.^43^ In this elaboration, the antibiotic prescriber is seen as responsible for the interests of *both* the patient and the wider community, which the ‘commons’ formulation necessarily places in opposition. When attempting to resolve this assumed conflict, and in concordance with dominant professional norms, clinicians often prioritize their immediate patient over the interests of other, distant and/or future patients.^32^^,^^33^^,^^44^ In addition to this bias, an increase in acquired ABR in the wider community is seen as a distant, and therefore dismissible, or at least discountable, concern.^45^^,^^46^

Although inescapable within the ‘commons’ formulation, this conflict of interest can disappear when the limitations of this formulation are considered. The most straightforward case is where antibiotic therapy offers no benefit at all (antibiotic abuse),^47^ yet this is the case in almost a third of all non-hospital antibiotic prescriptions and does not appear to be becoming less common in many settings.^48^ Slightly more complicated, although still free from conflict-of-interest challenges, is the situation where antibiotic therapy (or the specific prescription provided) does not offer a net benefit to the individual patient. Excessive antibiotic therapy, either in spectrum or duration (antibiotic misuse), appears to be the case in most prescriptions for conditions where narrower or shorter antibiotic therapy is beneficial.^17^^,^^49^ In such contexts, excessive therapy offers no net benefit and (depending on the adverse reaction profile of the chosen antibiotic) sometimes net harm.

It is thus a common situation that an antibiotic prescription is in the interest of neither the patient nor the wider community. This is a tragedy, but its structure is not that of the tragedy of the commons (wherein individual self-interest leads to collective ruin). The frequency of this problem seems to argue against random incompetence among prescribers and suggests the need for an expansion of the tally of the risk and benefits of these decisions to include the prescriber. Under this expansion, which can be labelled a ‘dual agent-principal’ problem, the prescriber can now be understood to be acting for the patient, the community and, also, ‘themselves’. Moral hazard arises when prescribers’ incentives do not align with those of the other two parties, and qualitative research has identified these adverse drivers of antibiotic prescribing as active in multiple medical contexts.^50^ Chief among these appear to be concerns regarding censure for ‘under-treatment’ relative to professional cultural norms (or even simply the established habits of senior colleagues^51^^,^^52^), independent of any effect on patient outcomes.^45^^,^^52^ Clinical uncertainty exacerbates the use of broad-spectrum agents, even though this same uncertainty reduces the probability that such an intervention is likely to be beneficial.^51^ At an extreme, these anxieties can culminate in fetishistic thinking not amenable to behavioural change via discussions of magnitude and distribution of risk.^53^ In some cases, where practitioners obtain financial gain by selling the antibiotics they prescribe, the incentive structure may be even more conflicted.

### Antimicrobial stewardship: promises and limitations

One potential response to the problems created by the conflicts of interest above is the appointment of ‘antibiotic stewards’—neutral professionals set apart from individual prescribers and their patients whose primary role is to steward the scarce resource of effective microbials by tipping the balance of average antibiotic use away from common instances of conflicted and profligate depletion. Yet this in turn raises the ancient dilemma, to paraphrase Plato: who will steward the stewards?

Agent-level conflicts affect those involved in stewardship teams.^54^ Clinicians in stewardship teams themselves will often be required to revise (and therefore implicitly criticize) the antibiotic plans instituted by their colleagues and may avoid recommending or enforcing optimal treatment plans in order to reduce real or perceived workplace conflicts.^53^ Although good working relationships might promote the longevity and thereby the long-term benefits of such person-to-person stewardship activities, the existence of such inextricable conflicts means that stewards will often fail to fulfil their primary role as neutral arbiters and points to the need for other policies to address inappropriate antibiotic use.

Moreover, stewardship resources are often concentrated in hospitals, where some of the more overt harms from resistant bacterial infections often manifest, but it is in the general community that the majority of antibiotic consumption occurs (with wide and unjustified variation in prescription rates per capita in otherwise similar communities^55^^,^^56^). Most acquired ABR is probably generated by this broad-based low-intensity use (for mild conditions where individual benefit is small) than by repeated use as often occurs in hospitalized patients.^57–59^

In this light, negotiating conflicts of, and conflicting, interests at a per-patient level looks to be far too narrow an approach. The scope of policy and intervention should arguably be much larger, addressing the more relevant and less ethically complicated phenomena of broad-based antibiotic abuse and misuse, and the factors that facilitate transmission of resistant pathogens between individuals.

## Other considerations

### Asymptomatic carriage

Public health measures to control antibiotic resistance raise ethical issues not only in cases where resistant bacteria cause harm to their hosts but also because public health measures may target the much larger number of healthy (asymptomatic) carriers of resistant organisms. The recent COVID-19 pandemic has highlighted the ethical salience of public health measures for asymptomatic infection, which sometimes involve infringements upon carriers’ freedoms and well-being in the name of reducing public health risks. Unlike infections with respiratory viruses that last days or weeks, resistant bacteria are often carried asymptomatically for years, meaning that cumulative infringements on carriers’ lives may be even more significant. Being identified as a carrier can result in restricted access to healthcare and mental health issues due to stigmatization,^60^^,^^61^ but also infringements on privacy, freedom of movement and free choice of occupation.^12^ Determining the conditions under which such infringements would be ethically justifiable, if any, requires not only more research to determine the risks that carriers face (due to resistant disease they might develop) and impose on others, but also careful ethical analysis regarding, for example, what degree of liberty infringement is justifiable for a given quantum of risk,^12^ and the extent to which carriage of resistant bacteria is more common in those facing (other) social disadvantages.

### Global distribution of resistance

**Fig. 1:**
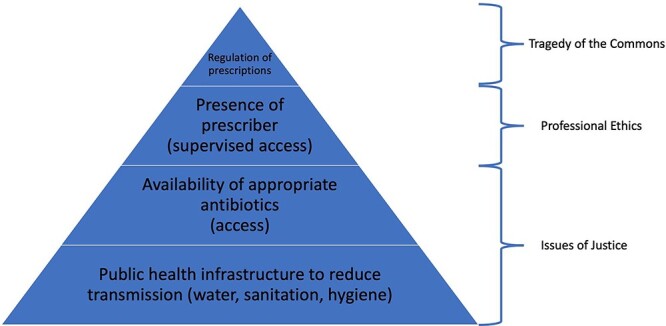
Global hierarchy of policy approaches for antibiotic resistance.

Much of the debate about antibiotic resistance focuses on ethically difficult trade-offs arising from antibiotic use, but these situations represent a tiny fraction of the global disease burden due to resistant bacteria. It has been noted that there is a stark difference between problems of ‘excess’ and those of ‘access’.^62^ While high-income countries struggle with the development of appropriate policies to curb inappropriate use of antibiotics available in abundance, hundreds of thousands of people die in low-income countries every year for want of access to antibiotics.^63^ Yet this contrast hides more basic problems of global health injustice in terms of the distribution of antibiotic resistance. Primordial prevention in the form of access to clean water, sanitation, basic hygiene and a minimum of economic prosperity is still unavailable to a large fraction of the global human population. Even before questions of whether people from such communities can access antibiotics and/or a healthcare provider to diagnose the disease and supervise access to antibiotics comes the need for basic public health measures (see Fig. 1). Not only do social determinants of health make infectious disease episodes more likely, they also promote the spread of resistant organisms (and resistance traits between organisms) as much of the environment in poor communities is contaminated with resistant bacteria.^9^ Without addressing these factors, stewardship efforts to reduce antibiotic use among the global poor (and, for that matter, in livestock and agriculture) will remain largely futile.

Above and beyond altruistic motivations for high-income countries to help address these social determinants in poor communities, there are also self-interested reasons: since resistant bacteria now travel easily from one community to another, and since containing an outbreak of highly resistant bacteria in high-income countries can exact a high toll in disease burden and economic costs, it is arguably in the self-interest of high-income countries to address the structural causes of global health injustice that contribute to the emergence, persistence, and spread of resistant bacteria.

## Future research and policy

The apparently deflationary conclusion that much of the framing of resistance policy has been oversimplified and/or misguided nevertheless opens the door to more innovative approaches in ethics, science and health policy. From an ethical perspective, policy and clinical decisions should be based on value judgements informed by sound evidence. Where this evidence is lacking there is an ethical imperative for more relevant scientific research and public health surveillance. Policy should also focus on harm reduction, and this in turn requires measuring harm.

Clinical science should therefore focus on producing evidence regarding (i) optimal antibiotic durations for common bacterial infections or criteria for antibiotic cessation (since most standard ‘courses’ of antibiotics appear excessive); (ii) the minimally effective antibiotic spectrum for specific types of infections, noting that the current paradigm of antimicrobial resistance testing may encourage unnecessary bystander selection^64^; (iii) non-antibiotic interventions against infection including vaccination, which itself can reduce new-onset and accumulated acquired ABR^2^^,^^65^; (iv) the direct harms of antibiotic therapy (e.g. determining the number needed to harm per dose or prescription, since these harms are often poorly characterised); (v) the more complex, potentially net harmful, longer-term effects of antibiotic use on the human microbiome at the individual and population level.

There is also a need for communities to define (via public engagement informed by clinical research and surveillance) locally acceptable antibiotic treatment thresholds, or the conditions under which not prescribing antibiotics of minor or marginal benefit would be socially acceptable. The resulting socially endorsed prescription policies would need to be updated as the local prevalence of resistance changes over time, and this in turn requires responsive public health surveillance systems that can feed back resistance data to policymakers and prescribers in a timely manner.

Ethics research might therefore focus on improving community engagement strategies, including how best to inform the public regarding the harms of antibiotic therapy, which appears to be an effective intervention to reduce consumption.^66^ More broadly, ethics work might focus on the justice implications of the global distribution of the harms of resistant infections, and the conditions under which (if any) public health measures might justifiably infringe on the freedoms and well-being of (asymptomatic) carriers of resistant pathogens under different prevalence scenarios. Finally, given the arguably unjustified devotion of enormous academic and public resources to studying COVID-19, ethicists should make the case for more appropriate priority setting, including the devotion of greater resources to responses to antibiotic resistance—a far more significant long-term problem for global public health than any novel virus to date.

## Conclusions

Antibiotic resistance and the use of antibiotics raise a range of ethical issues that have recently received more sustained analysis in the bioethics literature. Standard conceptual frameworks such as those related to patients’ moral responsibilities to comply with prescriptions, tragedies of the commons or stewardship have significant limitations and may distract from larger issues including the inequitable global distribution of resistant bacterial disease and access to antibiotics. Ethical analyses of public health control measures for antibiotic resistance should involve not only risk–benefit assessment but also consideration of how other ethical values such as fairness and liberty might be undermined or promoted. Strategies to improve the effectiveness of antimicrobial treatment, including through avoiding acquired ABR, must be based on a solid ethical and empirical foundation.

## Conflict of interest

The authors have no potential conflicts of interest.

## Data Availability

No new data were generated or analysed in support of this review.

## Funding

EJ is supported by Wellcome Trust (UK) [203132 and 221719].
